# Supplementary material for: Identification of photoperiod-induced specific miRNAs in the adrenal glands of Sunite sheep (Ovis aries)
Source: Front Vet Sci. 2022 Jul 22;9:888207. doi: 10.3389/fvets.2022.888207 (PMC9354845; doi:10.3389/fvets.2022.888207)
Supplement: Supplementary file 1 [file Data_Sheet_1.ZIP › Supplementary file/miRDeep2 file.zip!/miRDeep2 file/Novel_Structure/SP42A3/Novel_551.pdf]

Provisional ID	: NC_019460.2_7692
Score total	: 1.5
Score for star read(s)	: -1.3
Score for read counts	: 0
Score for mfe	: 1.2
Score for randfold	: 1.6
Score for cons. seed	:
Total read count	: 34
Mature read count	: 34
Loop read count	: 0
Star read count	: 0

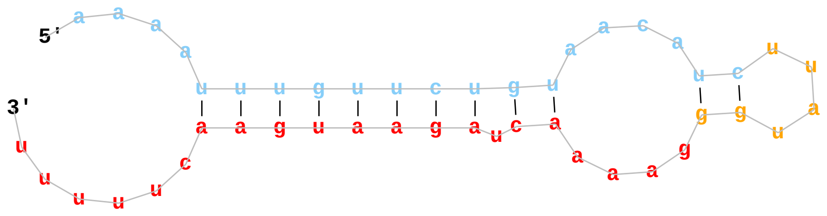

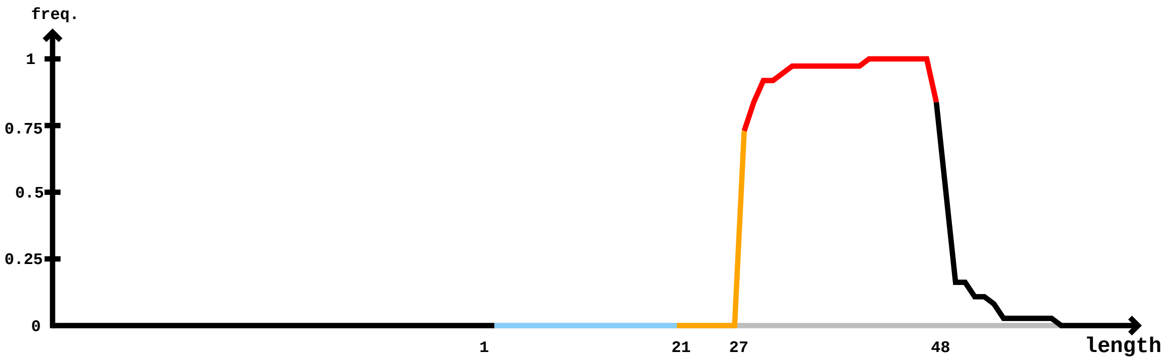

Star Mature

```
5'- ggggaagggugaaacagggcucucucuacuguugggcuggccagaaaauuuguucuguaacaucuuauggaaaaacuagaaugaacuuuuuggccaacucaguuauuucug   -3'    exp
    (((((.((((........)))))))))....(((((.((((((((((.((((((((((....((....))....)).)))))))).)))))))))).)))))........    reads    mm    sample
    ......................................................................Aaaaacuagaaugaacuuuu.....................    6        1     seq
    ......................................................................gaaaacuagaaugaacuuuuu....................    1        0     seq
    ......................................................................Aaaaacuagaaugaacuuuuu....................    17       1     seq
    ......................................................................Aaaaacuagaaugaacuuuuug...................    3        1     seq
    .......................................................................aaaacCagaaugaacuuuuu....................    3        1     seq
    .......................................................................aaaacuagaaugaacuuuuuggU.................    1        1     seq
    ........................................................................aaacCagaaugaacuuuuug...................    1        1     seq
    ........................................................................aaacuGgaaugaacuuuuuggc.................    1        1     seq
    ........................................................................aaacuUgaaugaacuuuuuggcca...............    1        1     seq
    ..........................................................................acuagaaugaacuuuuuggccaa..............    1        0     seq
    ...........................................................................cuCgaaugaacuuuuuggccaa..............    1        1     seq
    ...................................................................................aCcuuuuuggccaacucagu........    1        1     seq
```
